# Biopsychosocial Management of Rural Ankylosing Spondylitis in a Pregnant Woman: A Case Report

**DOI:** 10.7759/cureus.59187

**Published:** 2024-04-28

**Authors:** Ryuichi Ohta, Chiaki Sano

**Affiliations:** 1 Community Care, Unnan City Hospital, Unnan, JPN; 2 Community Medicine Management, Shimane University Faculty of Medicine, Izumo, JPN

**Keywords:** patient-centered care, breastfeeding, pain management, rural health services, ankylosing spondylitis, maternal health services

## Abstract

In a rural Japanese setting, this case report delves into managing a post-partum woman diagnosed with ankylosing spondyloarthritis (AS), showcasing the complexities of balancing effective pain relief with breastfeeding. The study highlights a multifaceted approach that incorporates medical treatment, psychosocial support, and comprehensive patient education, which are essential in rural healthcare where resources may be scarce. Initially managed with diclofenac due to its safer profile for breastfeeding, the patient's treatment was eventually escalated to adalimumab, aligning with improved circumstances regarding breastfeeding. This case emphasizes the critical role of holistic, patient-centered care in family medicine, particularly for managing maternal and child health chronic conditions. It illustrates how integrating mental health support, acknowledging patient fears, and educating families can significantly enhance patient care and outcomes. Through this approach, the report advocates for a broader application of family medicine principles to improve maternal and child health services in rural settings, demonstrating the importance of tailored healthcare strategies that consider patients' medical and emotional needs.

## Introduction

Maternal and child health (MCH) is essential in family medicine settings where family physicians deal with various health issues, from pediatrics to geriatrics [1]. Especially in rural family medicine, physicians need to deal with MCH issues related to complications such as rheumatic diseases [2]. In rural contexts, the lack of healthcare professionals and the multiple contraindications of medicine for rheumatic diseases can complicate the management of MCH [2]. Rural family physicians may need knowledge and skills to manage medical control of rheumatic diseases such as AS, while also respecting patients' perception regarding childbearing and the balance with the severity of their diseases [3,4]. This time, we had a young post-partum woman with the chief complaint of severe back pain that was impinging on her quality of life (QOL) [5]. She was diagnosed with AS and needs to discuss her treatment, considering the risk of the usage of rheumatic medicine for breastfeeding. This case report discusses effective treatments in family medicine using the framework of three-stage diagnosis respecting rural Japanese contexts.

## Case presentation

A 32-year-old female with para 1-0-0-1 came to the outpatient department of our rural community hospital with the chief complaints of gradually worsening back pain that was impinging on her QOL, such as walking, toileting, and taking a bath. When she was six months pregnant, she began to experience back pain that worsened in the morning. Her pain gradually worsened after the delivery, but it got better one month later. However, her pain worsened again three months after the delivery. Four months after the delivery, she was unable to walk or care for herself, so her husband brought her to our hospital. She did not have any symptoms, such as eye symptoms, peripheral joint pain, photosensitivity, oral ulcers, dysphagia, dyspnea, palpitation, and chest and abdominal pain.

Clinical perspective

The vital signs at the visit were as follows: blood pressure, 123/78 mmHg; pulse rate, 81 beats/min; body temperature, 36.5°C; respiratory rate, 18 breaths/min; and oxygen saturation, 97% on room air. Her physical examination revealed tenderness on the thoracic and lumbar vertebra, bilateral erector spinae, and sacroiliac joints. The range of motion of her back was limited to within 15° in flexion, extension, and rotation. There were no obvious abnormalities in the head, neck, chest, abdomen and skin. The laboratory data showed an elevated erythrocyte sedimentation rate without high titers of rheumatoid factor, anti-citrullinated protein antibodies, and antinuclear antibodies (Table 1).

**Table 1:** Initial laboratory data of the patient eGFR: Estimated glomerular filtration rate; CK: Creatine kinase; CRP: C-reactive protein; TSH: Thyroid-stimulating hormone; Ig: Immunoglobulin; HCV: Hepatitis C virus; SARS-CoV-2: Severe acute respiratory syndrome coronavirus 2; HIV: Human immunodeficiency virus; HBs: Hepatitis B surface antigen; HBc: Hepatitis B core antigen; S/CO: Sample/Cut-off; CCP: Cyclic citrullinated peptide.

Parameters	Level	Reference
White blood cells	6.8	3.5–9.1 × 10^3^/μL
Neutrophils	67.2	44.0%–72.0%
Lymphocytes	27.5	18.0%–59.0%
Monocytes	4.2	0.0%–12.0%
Eosinophils	0.6	0.0%–10.0%
Basophils	0.5	0.0%–3.0%
Red blood cells	4.58	3.76–5.50 × 10^6^/μL
Hemoglobin	13.3	11.3–15.2 g/dL
Hematocrit	40.2	33.4%–44.9%
Mean corpuscular volume	87.9	79.0–100.0 fl
Platelets	28.2	13.0–36.9 × 10^4^/μL
Erythrocyte sedimentation rate	35	2–10 mm/hour
Total protein	8.0	6.5–8.3 g/dL
Albumin	4.7	3.8–5.3 g/dL
Total bilirubin	0.5	0.2–1.2 mg/dL
Aspartate aminotransferase	20	8–38 IU/L
Alanine aminotransferase	18	4–43 IU/L
Alkaline phosphatase	158	38–113 U/L
γ-Glutamyl transpeptidase	12	<48 IU/L
Lactate dehydrogenase	134	121–245 U/L
Blood urea nitrogen	17.8	8–20 mg/dL
Creatinine	0.54	0.40–1.10 mg/dL
eGFR	90.0	>60.0 mL/min/L
Serum Na	141	135–150 mEq/L
Serum K	4.4	3.5–5.3 mEq/L
Serum Cl	103	98–110 mEq/L
Serum Ca	10.7	8.8–10.2 mg/dL
Serum P	3.8	2.7–4.6 mg/dL
Serum Mg	1.7	1.8–2.3 mg/dL
Ferritin	32.6	14.4–303.7 ng/mL
CK	75	56–244 U/L
CRP	0.34	<0.30 mg/dL
TSH	0.83	0.35–4.94 μIU/mL
Free T4	1.1	0.70–1.48 ng/dL
IgG	1364	870–1700 mg/dL
IgM	216	35–220 mg/dL
IgA	284	110–410 mg/dL
IgE	113	<173 mg/dL
HBs antigen	0.0	IU/mL
HBs antibody	0.00	mIU/mL
HBc antibody	0.00	S/CO
HCV antibody	0.00	S/CO
Syphilis treponema antibody	0.00	S/CO
SARS-CoV-2 antigen	Negative	Negative
Antinuclear antibody	40	<40
Homogeneous	40	<40
Speckled	40	<40
Anti-CCP antibody	<0.6	<5 U/ml
Urine test		
Leukocyte	Negative	Negative
Nitrite	Negative	Negative
Protein	Negative	Negative
Glucose	Negative	Negative
Urobilinogen	Negative	Negative
Bilirubin	Negative	Negative
Ketone	Negative	Negative
Blood	Negative	Negative
pH	7.0	-
Specific gravity	1.012	-

**Figure 1:**
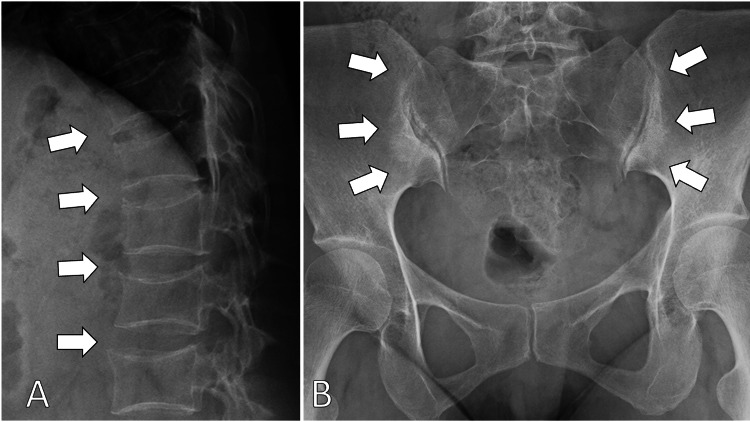
X-ray of the back (A) and pelvis (B) showing multiple bone spurs in the thoracic and lumbar vertebrae and the bilateral deformation of sacroiliac joints (white arrows)

X-ray findings of the spine showed multiple bone spurs in the thoracic and lumbar vertebrae and the bilateral deformation of sacroiliac joints (Figure 1).

**Figure 2:**
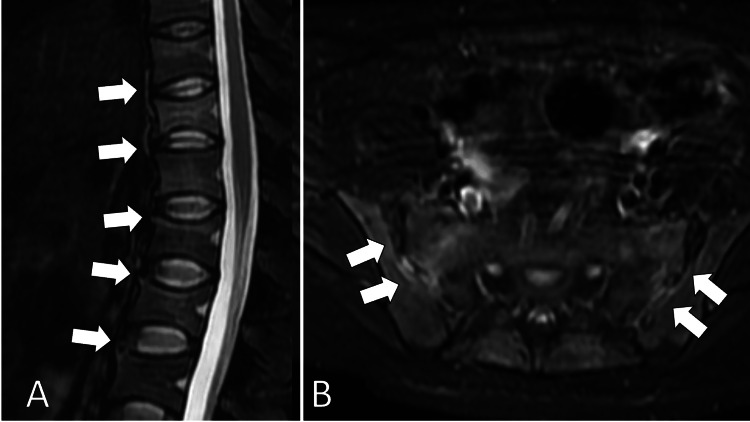
Magnetic resonance imaging of the thoracic and lumbar vertebra (A) and pelvis (B) confirming the inflammation of the vertebral and sacroiliac joints (white arrows)

MRI of the thoracic and lumbar vertebra and pelvis confirmed the inflammation of the vertebral and sacroiliac joints (Figure 2).

Clinically, she was diagnosed with the early phase of ankylosing spondyloarthritis (AS) [6]. She was under medical care with prompt treatment involving immunosuppressant medications.

Individual aspect

Her pain from AS had a significant impact on her life, and she was unable to walk, bathe, and change her clothes on her own. She feared the continuity and progressiveness of the pain for her life as well as the possibility of becoming permanently dependent on her family. As she observed some AS patients, she was worried about becoming like them. Consequently, she experienced depression and loss of motivation for childbearing. She was also anxious about the breastfeeding difficulties caused by the treatment of AS with immunosuppressants, which could affect child growth [6]. Her treatment expectations encompassed effective pain management, the resumption of appropriate childbearing, and reducing the risk of breastfeeding complications.

Contextual dimension

The patient lived with her husband, six-month-old son, and parents. She worked as a medical clerk in our hospital and supported in our clerical work. With her husband working during the daytime and her retiring parents available for assistance, she could come to our hospital for care. She did not have any financial issues to receive treatment for AS. Her six-month-old son was the first baby in her family. Her family members tried to bear the son without any difficulties, so they were anxious about the future of her childbearing. Her family also hoped for effective treatment with fewer breastfeeding-related side effects. These contextual factors significantly influenced her treatment and management strategy. In addition, while working at the hospital before maternity leave, the patient inquired about her back pain. As we did not perceive her condition as critical, we did not recommend her to visit our outpatient department.

Treatment and response

Following a discussion with the patient and her husband about the treatment options, a daily dose of 75 mg diclofenac was started, considering the risk of side effects on breastfeeding and childbearing. During the follow-up in two weeks, her back and pelvic pain was alleviated, and she could walk independently and care for herself. However, the morning stiffness of the back was persistent. Although the usage of tumor necrosing factor inhibitors may be necessary to prevent AS progression, she wanted to bear her son by breastfeeding who was unable to drink infant formula [6]. After two months of diclofenac treatment, her son could drink infant formula; so, she decided to use intramuscular adalimumab of 40 mg biweekly. After starting adalimumab, the morning stiffness of her back disappeared, allowing her to lead her life independently and enjoy childbearing.

## Discussion

This case study highlights the critical role of family medicine principles in addressing complex MCH issues, particularly in rural settings. The holistic approach of family medicine encompasses the biopsychosocial model and is essential in managing conditions like AS during motherhood [7]. In rural healthcare settings, where resources are often limited, the comprehensive nature of family medicine becomes even more significant [8]. The case of the young post-partum woman with AS illuminates this point. The management of her condition required not only an understanding of the medical aspects of AS but also a deep appreciation of the psychosocial and emotional challenges faced by a new mother coping with a chronic disease.

The holistic approach of family medicine, embracing the biopsychosocial model, is indispensable for managing chronic conditions like AS during motherhood, especially in rural healthcare settings where resources are often limited [7,8]. This case highlights the complexity of addressing medical and psychosocial challenges faced by a new mother with AS, showcasing the essential role of family medicine's comprehensive approach in navigating the multifaceted nature of MCH.

Patient-centered care, a cornerstone of family medicine, was crucial in managing this case [9]. Addressing the patient's concerns about the impact of her medication on breastfeeding and her ability to care for her child underscores the importance of incorporating patient preferences, needs, and values into their care plans. This individualized treatment strategy, particularly relevant in rural settings, underscores the efficacy of patient-centered approaches in alleviating patient and family anxieties related to childbearing and medical issues. Our approach to managing this patient's condition underlines the need for individualized treatment strategies in MCH, considering the unique circumstances of each patient. In rural contexts such as this case, patients and families may be anxious about childbearing and related medical issues. Patient-centered approaches in rural contexts can mitigate their anxieties.

The profound psychological impact of chronic diseases like AS, especially in the post-partum period, highlights the importance of integrating mental health care into MCH [10]. Family physicians are uniquely positioned to address these concerns due to their ongoing relationship with patients and families. This case illustrates the necessity of an integrated care approach that addresses physical and mental health, emphasizing the critical role of mental health support and education within family medicine to enhance MCH outcomes [11]. Addressing these concerns was as crucial as managing her AS, demonstrating the importance of an integrated approach to MCH in family medicine. Understanding the connection between physical and mental conditions in MCH in rural communities can improve mental conditions and should be educated in family medicine education in Japan [10].

Education is a critical component of family medicine, where physicians often serve as educators for their patients and families [12]. In this case, patient and family education about AS and its implications for breastfeeding and childcare empowered informed decision-making, demonstrating the value of education in MCH. Reflection and continuous improvement, as facilitated by Gibbs' Reflective Cycle, highlight the importance of vigilance and early intervention in MCH, emphasizing the educational role of family physicians in rural healthcare settings [13]. Concretely, respecting rural conditions, rural family physicians should be educated about MCH and approach communities for continual education about it.

The insights from this case have profound implications for future MCH management in rural settings [14]. This case highlights the complexity of managing chronic conditions like AS in the context of MCH. It underscores the need for holistic care that considers the patient's role as a mother and the implications of treatment on family life [15]. They advocate for a vigilant approach to early symptom recognition, comprehensive education for patients and families, and an integrated mental health care strategy. This case reinforces the need for a holistic, family-centered approach in MCH, particularly for managing chronic conditions like AS in the context of motherhood [16]. It highlights the crucial role of family medicine principles in improving patient care and outcomes in rural healthcare environments [17]. The insights gained from this case have profound implications for future MCH management in rural settings. A more vigilant approach to early symptoms, comprehensive patient education, and deeper integration of mental health care are aspects that we plan to strengthen in our practice [14]. This case underscores the necessity of adopting a holistic, family-centered approach in MCH, particularly in managing chronic conditions like AS in the context of motherhood.

## Conclusions

In conclusion, this case report reaffirms the indispensable role of family medicine principles in MCH. The holistic, patient-centered approach, integral to family medicine, is crucial in managing complex health conditions in motherhood, especially in resource-limited rural settings. This approach ensures comprehensive medical care and addresses the broader psychosocial and educational needs of patients and their families, paving the way for more effective and empathetic healthcare in MCH. A more thorough assessment of her symptoms before her maternity leave could have led to an earlier diagnosis. Also, exploring alternative pain management strategies that are compatible with breastfeeding earlier might have been beneficial. While considering disease control, patient, and family-centered approaches, the involvement of families in the discussion of treatment is vital for MCH care in rural contexts where healthcare resources are lacking.
